# Co-Designing, Developing, and Testing a Mental Health Platform for Young People Using a Participatory Design Methodology in Colombia: Mixed Methods Study

**DOI:** 10.2196/66558

**Published:** 2025-05-06

**Authors:** Laura Ospina-Pinillos, Débora L Shambo-Rodríguez, Mónica Natalí Sánchez-Nítola, Alexandra Morales, Laura C Gallego-Sanchez, María Isabel Riaño-Fonseca, Andrea Carolina Bello-Tocancipá, Alvaro Navarro-Mancilla, Jaime A Pavlich-Mariscal, Alexandra Pomares-Quimbaya, Carlos Gómez-Restrepo, Ian B Hickie, Jo-An Occhipinti

**Affiliations:** 1 Department of Psychiatry and Mental Health School of Medicine Pontificia Universidad Javeriana Bogota Colombia; 2 Keralty Medical Centers Bogota Colombia; 3 Medicalnet SAS Bogota Colombia; 4 Department of Systems Engineering School of Engineering Pontificia Universidad Javeriana Bogota Colombia; 5 Department of Clinical Epidemiology and Biostatistics School of Medicine Pontificia Universidad Javeriana Bogota Colombia; 6 Faculty of Medicine and Health The University of Sydney Sydney Australia; 7 Mental Wealth Initiative, Brain and Mind Centre Faculty of Medicine and Health The University of Sydney Sydney Australia; 8 Computer Simulation and Advanced Research Technologies (CSART) Sydney Australia

**Keywords:** low- and middle-income countries, Latin America, telemedicine, medical informatics, eHealth, mental health, help seeking, community-based participatory research, patient participation, patient satisfaction

## Abstract

**Background:**

Globally, mental health (MH) problems increasingly affect young people, contributing significantly to disability and disease. In low- and middle-income countries, such as Colombia, barriers to accessing care exacerbate the treatment gap. In addition, the lack of widespread digital interventions further deepens the digital health divide between the Global North and Global South, limiting equitable access to innovative MH solutions.

**Objective:**

This study aims to co-design and develop an MH platform using participatory design methodologies and conduct a 15-month naturalistic observational trial to assess its feasibility among Colombian youth.

**Methods:**

This study used a mixed methods approach within a structured research and development cycle. To ensure a user-centered design, we began with a series of co-design workshops, where stakeholders collaboratively identified key user needs. Following this, usability testing was conducted in 2 stages, alpha and beta, using the System Usability Scale (SUS) to assess functionality and user experience. To capture real-world interactions, a naturalistic observational trial ran from July 2022 to October 2023, collecting data on user engagement and system performance. This study integrated quantitative and qualitative analyses.

**Results:**

A total of 146 individuals participated in the co-design process, with 110 (75.3%) contributing to the development of platform components and 36 (24.7%) participating in usability testing. The co-designed platform integrated several key features, including social media and advertising, an MH screening tool, registration, targeted psychoeducational resources, automated tailored recommendations, and a “track-as-you-go” feature for continuous MH monitoring. Additional elements included user-friendly follow-up graphs, telecounseling integration, customizable well-being nudges, an emergency button, and gamification components to enhance engagement. During usability testing, the beta prototype received a median SUS score of 85.0 (IQR 80-92.5), indicating high usability. In the subsequent observational trial, which ran from July 2022 to October 2023, a total of 435 users interacted with the platform—314 (72.2%) as registered users and 121 (27.8%) anonymously. Emotional distress was prevalent, with 63.7% (200/314) of the registered users and 61.2% (74/121) of the anonymous users reporting distress, as measured by the 6-item Kessler Psychological Distress Scale. Despite 102 users requesting telecounseling, only 26.5% (27/102) completed a session. While usability scores remained high, engagement challenges emerged, with only 18.8% (59/314) of the users continuing platform use beyond the first day.

**Conclusions:**

This study explored the development and user experience of a youth MH platform in Colombia, demonstrating that a cocreation approach is both feasible and effective. By actively involving users throughout the design process, the platform achieved high usability and incorporated features that resonated with its target audience. However, sustaining long-term engagement remains a challenge, as does addressing privacy concerns, particularly for younger users. These findings highlight the importance of continuous user-centered refinement to enhance both accessibility and retention in digital MH interventions.

## Introduction

### Background

Globally, 1 in 7 young people aged 10 to 19 years are estimated to experience a mental illness [1]. Depression, anxiety, and behavioral disorders are considered some of the main contributors to the burden of disability and disease in young populations [2,3]. Likewise, suicide has been found to be one of the main causes of death in this population [1,4]. Unsurprisingly, the economic and human costs of mental ill-health represent a public health concern where urgent actions are required to improve health outcomes, reduce years of life lost due to preventable mortality and disability, and save millions in health care spending [4]. The impacts of mental ill-health are particularly challenging for low- and middle-income countries, which are home to 90% of the world’s youth [5].

Evidence suggests that there can be up to a decade-long delay between the first symptoms of a mental health (MH) disorder and initial contact with services, especially among young people [6]; consequently, the use of digital health tools (DHTs) has gained attention as a potential solution, as young people often seek information online about their MH before discussing it with family members or engaging with MH services [7]. Young people have a preference to find a middle ground between formal and informal sources of information, which opens the opportunity to engage them with MH services or with sources of care [8]. Notwithstanding the value of DHTs, challenges to their adoption exist. First, the internet and digital technologies might not be equally accessible across and within countries [9,10]. Second, even in countries where DHTs are readily available, retention of users and usability are still their main challenges, as it is reported that their use decreases throughout the trials, and most evaluations are limited to short periods [11-13]. To promote the adoption of DHTs [14], participatory design approaches [15,16] offer a promising solution by centering users’ needs. This user-driven methodology not only enhances usability but also fosters long-term engagement, ensuring that the technology remains relevant and effectively integrated into everyday practice [17].

Colombia is an upper–middle-income country [18] that faces many societal and economic challenges, further exacerbated by the COVID-19 pandemic [18]. The latest MH survey indicates that 26.5% of adolescents and 26.2% of adults aged 18 to 44 years are likely to experience >2 MH problems [19]. Despite this high prevalence, service use remains low, with only 12.4% of the individuals in urban areas with MH disorders having accessed any health services, according to the World Health Organization [20]. The substantial treatment gap in MH care underscores the critical need for enhanced access to services, particularly given the high prevalence of multiple MH issues among adolescents and young adults. This gap is intrinsically linked to the help-seeking process, as barriers, such as stigma, lack of awareness, and limited availability of resources, prevent individuals from seeking and receiving necessary support [21].

### This Study

To the best of our knowledge, there are no comprehensive DHTs available in Bogotá, the capital city, or elsewhere in Colombia that assist young people with their MH. Using participatory design methodologies with end users, this research study explored the ideal design of an MH platform for young people in Colombia, with a focus on essential functionalities and visual appearance to effectively address their MH needs and preferences. We aimed to describe the co-design process and outcomes, outline the development process, and conduct preliminary testing of the minimum viable product (MVP) of this youth MH platform.

## Methods

### Overview

In line with the World Health Organization framework for developing digital interventions [22], through a well-tested research and development (R&D) cycle [15,23,24], a youth MH platform was co-designed, developed, and tested.

Recruitment involved convenience and snowball sampling, using peer groups and advisory representatives. Participants represented end users of the platform (young people, people with lived experience, their supportive others [eg, carers, family, friends, and educators], and health professionals [eg, MH professionals and allied health professionals]).

### Ethical Considerations

The study was approved by the human research ethics committee of Pontificia Universidad Javeriana and Hospital Universitario San Ignacio (protocol number FM-CIE-0103-21). Using REDCap (Research Electronic Data Capture; Vanderbilt University), informed consent was obtained from adult participants and legal representatives of minors, who also provided their informed assent, as per national regulations. Data were deidentified and safely stored in institutional cloud servers (OneDrive), only accessible to the principal investigator and research officers. Participants in the study received a grocery store voucher in recognition for their time and expertise. For codesign workshops, nonmedical staff and community participants (including young people, people with lived experience, and their supportive others) received a COP $50,000 voucher, while health professionals who participated outside of regular working hours received a COP $100,000 voucher. For user testing sessions, the same groups received COP $40,000 and COP $60,000 Colombian, respectively. These differences in voucher amounts reflected the varying levels of time and effort required for each activity (COP $1000=US $0.26, on average, during project activities).

### Co-Design

Co-design workshops (CDWs) aimed to determine the needs and wants of end users as well as understand the functionalities and interface ideas they had for an MH platform. The information collected in these workshops was used to shape the actual development of the platform. What was developed and what it could look like was first determined in a knowledge translation session held after the workshops. The meeting included a multidisciplinary team composed of researchers, psychologists, psychiatrists, legal team members, engineers, and young people and representatives of people with lived experience. Mock-ups were then iteratively refined across each workshop. Furthermore, in between these meetings, several sessions with the reference group were held to always have the end users engaged in the decision-making process.

The CDWs were run using the Google Meet platform [25] in a 2-hour period with around 6 to 8 participants. Information collection was undertaken via surveys, word clouds, Cartesian planes, sketching pads, and scribble sheets [25].

Qualitative data were analyzed using NVivo (Lumivero) and a thematic analysis framework with deductive and inductive coding. An iterative approach guided each phase, and consensus methods were applied at the end of each phase (eg, counts and frequencies). Coding frequencies (CFs) were calculated for all subcategories within the main themes. Interrater reliability was measured using NVivo’s coding comparison feature, with 2 researchers independently coding CDW transcriptions until achieving at least a fair agreement based on Cohen κ statistic [26]. Once reliability was reached, the remaining material was individually coded.

### Development

Guided by young people’s wants and needs and considerations from the multidisciplinary team, several software prototypes were developed as responsive web applications for mobile and desktop devices. Considerations included the assessment of technical feasibility, resource availability, complexity, prioritization, and compliance requirements, among others.

Usability testing was divided into 2 stages. The alpha prototype testing involved 90-minute, one-on-one sessions using a think-aloud protocol, with task difficulty measured by the Single Ease Question scale [27]. Following this, feedback on corrections and reasonable adjustments was provided to the engineering team for the beta prototype. As this version offered a more refined MVP, beta testing followed the same procedures as the alpha sessions but included a more detailed usability evaluation using the System Usability Scale [28].

For data analysis of user testing, sets of notes were generated for each session according to the interviewer’s guide. Each section of all sets of notes was read for the first time to identify common themes or recommendations made by participants. Once themes were identified, sections were examined a second time to calculate frequencies (ie, to identify how many participants gave the same recommendation or expressed a similar opinion). Careful attention was given to negative or divergent opinions, for which the number of occurrences was also calculated. This process was carried out for each section of the notes until all sections were completed. Notes from alpha tests were examined first, followed by notes from beta tests. Microsoft Excel was used for frequency calculation.

### Testing

To test the MVP, a naturalistic trial was run from July 2022 to October 2023, collecting data on use in real-world settings, including feature use, survey responses, and sociodemographic information.

The quantitative analysis used descriptive statistics to summarize the data. For skewed distributions, the median was used as the preferred measure of central tendency. Variability was assessed through SDs and IQRs, while frequencies and percentages were used to summarize categorical variables. The relationships between categorical variables were analyzed using the chi-square test with a 95% CI. The analysis was conducted using SPSS (version 23; IBM Corp) and Microsoft Excel.

## Results

### Overview

A total of 146 participants were part of the co-design (n=110, 75.3%) and development (n=36, 24.7%) of the platform’s MVP. Most participants were female (n=103, 70.5%), with a median age of 18 (IQR 16-29) years. Middle (tiers 3 and 4) and low (tiers 1 and 2) socioeconomic tiers were the most represented in the sample, with 130 (89%) individuals. Vulnerable populations, such as survivors of armed conflict (n=6, 4.1%), ethnic minority individuals (n=5, 3.4%), and people with disabilities (n=1, 0.7%), were also part of the study.

### Co-Design

#### Overview

A total of 19 CDWs were run, 125 source documents were collected, and 1309 annotations were coded. The deductive coding included 3 themes based on the framework by Ospina-Pinillos et al [15,23]: functionality (actions that can be performed within the platform; annotations: n=504, 38.5%, including all subcategories), user interface (annotations: n=420, 32.09%), and privacy and data management (annotations: n=86, 6.57%). In total, 2 themes emerged, namely technology use for health (annotations: n=111, 8.48%) and youth MH problems (annotations: n=188, 14.36%). Interrater reliability demonstrated a moderate agreement (Cohen κ=0.490).

Results on the use of technology for health and youth MH problems, presented subsequently, highlighted the potential of a technological platform for young people and provided key insights for the development stage. Specific wants and needs regarding functionality, user interface, privacy, and data management are also described subsequently.

#### Technology Use for Health

Most participants said they used the internet for information seeking (eg, symptoms, tips for managing health concerns, information about healthy lifestyles, and reviews about health professionals), teleconsultation (both from the health care provider and patient perspectives), education, and low intensity treatments (yoga, meditation, and breathing exercises). Information was obtained both from formal sources (eg, e-books) and informal sources, including peer-generated information, influencers, and social media. Acknowledging the amount of misinformation on the internet, participants mentioned developing diverse techniques to triangulate the veracity of the information.

#### Youth MH Problems

During workshops, each participant contributed to word clouds of youth MH problems, which were used to identify 123 concerns and guide platform development. The most frequently mentioned difficulties, shown in Table 1, involved social relations (n=39, 31.7% mentions), followed by MH disorders (n=28, 22.8% mentions) and problems with self-esteem and self-concept (n=23, 18.7% mentions).

**Table 1:** Most frequently mentioned youth mental health problems in Bogotá, as reported by co-design workshop participants (N=123).

Category			Mentions, n (%)
**Problems with social relationships**			39 (31.7)
	Family problems	10 (8.1)	
	Peer problems	8 (6.5)	
	Bullying (including cyberbullying)	3 (2.4)	
	Couple problems	2 (1.6)	
	Others	16 (13)	
**MH^a^ disorders**			28 (22.8)
	Substance use	9 (7.3)	
	Depression	9 (7.3)	
	Anxiety	6 (4.9)	
	Eating disorders	2 (1.6)	
	Attention deficit	2 (1.6)	
**Problems with self-esteem and self-concept**			23 (18.7)
	Self-esteem	10 (8.1)	
	Self-concept	7 (5.7)	
	Life project	6 (4.9)	
**Emotional difficulties**			16 (13)
	Emotion regulation	4 (3.3)	
	Loneliness	4 (3.3)	
	Stress	3 (2.4)	
	Resilience	1 (0.8)	
	Recklessness	1 (0.8)	
	Hope	1 (0.8)	
	Boredom	1 (0.8)	
	Sadness	1 (0.8)	
Sexual health			5 (4.1)
Economic difficulties			5 (4.1)
Self-harm and suicide			3 (2.4)
Academic difficulties			3 (2.4)
Pandemic			1 (0.8)

^a^MH: mental health.

#### Functionality

This theme included 12 subcategories, for which we calculated the CF.

##### Reliable Information

Participants wanted to receive reliable, targeted, customizable, and periodically updated information (annotations: 52/504, CF=10.3%) delivered in multimedia format. They suggested adapting the platform to users’ needs by including an initial test to personalize content and featuring an animated character to guide users and deliver information.

In terms of topics, participants believed the platform should provide general information about MH (eg, warning signs); specific recommendations for healthy lifestyles (eg, nutrition); instructions or tutorials for mental hygiene activities (eg, breathing exercises); and infographics, worksheets, and information on leisure activities (eg, hiking groups). Where relevant, the platform should also redirect users to other, more specialized resources (eg, fitness apps). Information about helplines, spirituality, alcohol and drugs, as well as sexual health was also mentioned. For informative pieces on high-risk topics, such as sexual abuse or suicidal ideation, allied health and MH professionals stated that clear information should be provided about the formal service routes available in Colombia.

##### Registration

Participants highlighted the value of choosing to explore the platform anonymously or creating an account (annotations: 36/504, CF=7.1%). They proposed to have different user profiles (eg, young people, health professionals, and caretakers), which would receive targeted content.

They proposed the inclusion of different user profiles (eg, young people, health professionals, and caretakers), acknowledging that a young person’s MH care benefits from a collaborative support system involving both school and family. By incorporating multiple profiles, the platform would facilitate better interaction and coordination among these key stakeholders, ensuring more comprehensive support for young people:

I would create the account so that, let’s say, you would have more options, right? Because if you say, “Well, I’m a woman, a teenager, a girl,” it would give you more tailored options, right?

Adolescent from a CDW

##### Virtual Care

Participants emphasized the importance of being heard, suggesting the platform should offer options for discussing MH with professionals via videoconference, call, or chat while also highlighting the need for both crisis support and follow-up care (annotations: 33/504, CF=6.5%).

Participants expressed a desire for counseling, guidance, and practical advice tailored to their concerns, with some suggesting virtual care should include professional-led activities such as meditation or life project discussions:

I liked the idea of the calls because something similar was done in another project. Two young psychology students would call us monthly or weekly, depending on our schedule, to check in on how we were doing. They even had a project to help us create a life plan, guiding us on what we wanted and providing orientation.

Adolescent from a CDW

Others preferred simpler interventions, such as having professionals available to answer specific questions. A blended approach, combining virtual care with face-to-face interactions for initial meetings or home visits, was recommended by some to build rapport and foster empathy.

##### Screening Tests and Continuous Monitoring of MH Areas

Participants prioritized the ability to evaluate their well-being through tests (annotations: 153/504, CF=30.4%) with immediate results to track progress and express their feelings:

It works well because it might help the person express how they’re feeling at that moment. Maybe they don’t have someone to talk to and share those feelings, like saying, “I haven’t been sleeping well” or “I’m feeling sad” or “This happened to me yesterday.” It’s a way to express yourself while also getting help to feel better and reflect, asking yourself, “What can I do to sleep better?” I’d say it’s a good thing.

Adolescent from CDW

They wanted diverse response formats (eg, text, video, and audio), customizable survey reminders, and coverage of various topics, such as loneliness, physical health, and emotions. MH professionals suggested tracking coping strategies and integrating the platform into therapy. They emphasized that test results should include actionable feedback or resource links to support improvement or maintain progress.

##### Gamification

Participants emphasized the value of gamification (annotation: 42/504, CF=8.3%) to engage and motivate young people, suggesting elements, such as video games, avatars, digital medals, and incentives, such as vouchers, or proposing combinations, such as avatars, integrated into games or tied to rewards.

##### Social Media and Advertising

Participants suggested integrating the platform with social media (annotations: 68/504, CF=13.5%) through visually appealing and relatable advertisements on platforms, such as Facebook, Instagram, TikTok, and Twitter (subsequently rebranded X), focusing on common concerns, such as sleep and mood. They proposed using hashtags for awareness campaigns, adding a share button for easy promotion, and collaborating with influencers or YouTubers to donate content to engage young people and normalize MH concerns.

##### Well-Being Nudges

Participants acknowledged the diversity of MH concerns and coping strategies among young people. Suggestions included a calendar to track results, a virtual trainer to encourage engagement in activities, and notifications with motivational messages and activity reminders (annotations: 36/504, CF=7.1%).

##### Emergencies

Participants expressed a desire to either talk to someone immediately or receive guidance from the platform, but they also wanted the option to act independently or support a friend in need. In emergency situations, they emphasized the importance of quickly accessing help (annotations: 3/504, CF=0.6%). In addition, they suggested the ability to notify the platform if another user appeared to require urgent care.

##### Community Participation

Participants suggested adding blogs and forums (annotations: 60/504, CF=11.9%) moderated by health professionals for safety. They wanted to discuss MH and shared interests, such as soccer or movies, emphasizing that interaction with others could foster support, bonding, and mood improvement.

##### Chatbot

Participants suggested including artificial intelligence (annotations: 6/504, CF=1.2%) in the platform to provide care during after-hours or weekends, offering general advice, or linking users to specialized resources. They noted that chatbots could also benefit introverted users who may find it difficult to share their concerns.

##### Interoperability With Local Services

Participants recommended integrating the platform with local services (annotations: 12/504, CF=2.4%) either by referring users to specialized city services or incorporating it into existing programs offered by private and public organizations.

##### Research and Public Health Surveillance

Participants, particularly MH and allied health professionals, highlighted the research and public health value of an MH platform (annotations: 3/504, CF=0.6%). They suggested using data from continuous monitoring for epidemiological purposes to improve care for young people and integrating it into health surveillance systems.

#### User Interface

User interface refers to the visual presentation of content and functionalities. Participants stated that the platform should be visually appealing, engaging, and responsive, using inclusive language. Lay terms were preferred over clinical jargon. Customization and interactivity were essential characteristics to be considered across all platform functionalities.

The most important aspect for end users was an eye-catching, aesthetically pleasing appearance. Participants consistently mentioned using images, cartoons, and icons but emphasized that the platform should not appear childish.

#### Privacy and Data Management

Regarding data management, participants preferred clear, concise information about how their data would be used, favoring simple language over lengthy, jargon-filled documents while still adhering to privacy standards. Concerns were raised about obtaining consent for children aged <14 years, who could not legally provide consent in Colombia. While some younger participants were open to involving their legal guardians, most preferred to engage independently without parental authorization. In addition, most parents expressed a desire to access their children’s data:

If she were to use the platform, I would want to know absolutely everything—the good, the risky, the threatening, the happy—everything.

Parent from a CDW

They acknowledged that children may be hesitant to share their concerns if parents had access to the data, and although most would allow them to use the platform, for some, it could be a sign of poor or declining MH. Some parents indicated they would like to use the platform too, which showcased the multiple ways in which they wished to be involved:

Each child is a different world, everyone has their own way of thinking, and not everything in life is complete. I believe that both children and us as parents have our flaws or gaps. When looking at the platform, I think it would be great to use it to explore those gaps or the things we sometimes don’t understand about them or wish we could know.

Parent from a CDW

### Development

#### Functionality

##### Reliable Information

To expand access to Spanish-language psychoeducation resources, we integrated our platform with Mental Punto de Apoyo [29], the largest repository of MH materials in the region. This integration allows users to explore a wide range of resources, including videos, pamphlets, and podcasts on MH topics, along with practical techniques to enhance well-being (eg, meditation).

On our platform, these resources are presented with their title and images. When users click on an image, they are redirected to the Mental Punto de Apoyo website for full access. The repository is regularly updated, ensuring users have access to the latest information.

In addition, registered users benefit from tailored recommendations based on periodic survey results and can track a chronological record of all suggested resources, strengthening the connection between both platforms and enhancing the user experience.

##### Registration

We developed a registration feature that allows users to either create an account or continue anonymously. Anonymous users can access general MH resources but must provide basic demographic details and accept the terms of use. However, registered users gain access to additional features on their home page, including an SOS button, a customizable well-being plan, and avatar selection.

During development, parents and caregivers expressed interest in having their own profiles to better support their children. However, this feature was not included in the MVP.

##### Virtual Care

To provide access to virtual care, we integrated the platform with Mentes Colectivas, a free web-based program providing counseling and support for individuals aged ≥14 years. The program, accessible on a website [30], offers a safe space where users can seek guidance on emotional well-being, sexuality, and family planning. Users can connect with a team of students, professionals, and volunteers through their preferred digital contact method—telephone call, web-based chat, or web-based call. Appointments can be scheduled within 72 hours, with up to 5 follow-up sessions available as needed. For emotional well-being counseling, support is provided directly through the platform, including follow-up sessions. Users requiring urgent assistance outside of service hours are redirected to emergency services. For sexuality and family planning counseling, users are seamlessly transferred to a designated website using their platform credentials, with unlimited follow-up sessions available if needed [31].

##### Screening Tests and Continuous Monitoring of MH Areas

An MH screening and continuous monitoring feature (“track-as-you-go” functionality) was developed based on key concerns identified by CDW participants (Table 1). First-time users, whether anonymous or registered, complete an initial screener using the survey by Kessler et al [32] to assess concerns and distress levels, receiving tailored feedback and psychoeducation through automated algorithms. Registered users can access the continuous monitoring function, selecting from 13 areas (stress, loneliness, well-being, emotions, physical activity, sleep, subjective health status, self-esteem, relationships, eating, alcohol and substance use, bullying, and sexual and reproductive health) to track their progress. Surveys can be scheduled daily, weekly, or biweekly, with a maximum of 2 available at a time to encourage mindful participation. Registered users also gain access to visual progress charts, including line graphs, gauges, and calendars, with options to filter results by date, view them alongside resource histories, and export them as PDFs.

##### Gamification

During knowledge translation sessions, we recognized the importance of gamification in enhancing user engagement. However, due to the complexity of developing video games and incentive systems, we focused on implementing avatars as the first step. Users can choose from a set of predefined avatars and update them at any time, allowing a degree of personalization. While this feature laid the foundation for a more interactive experience, future iterations may explore additional gamification elements to further enhance user motivation and engagement.

##### Social Media and Advertising

**Figure 1:**
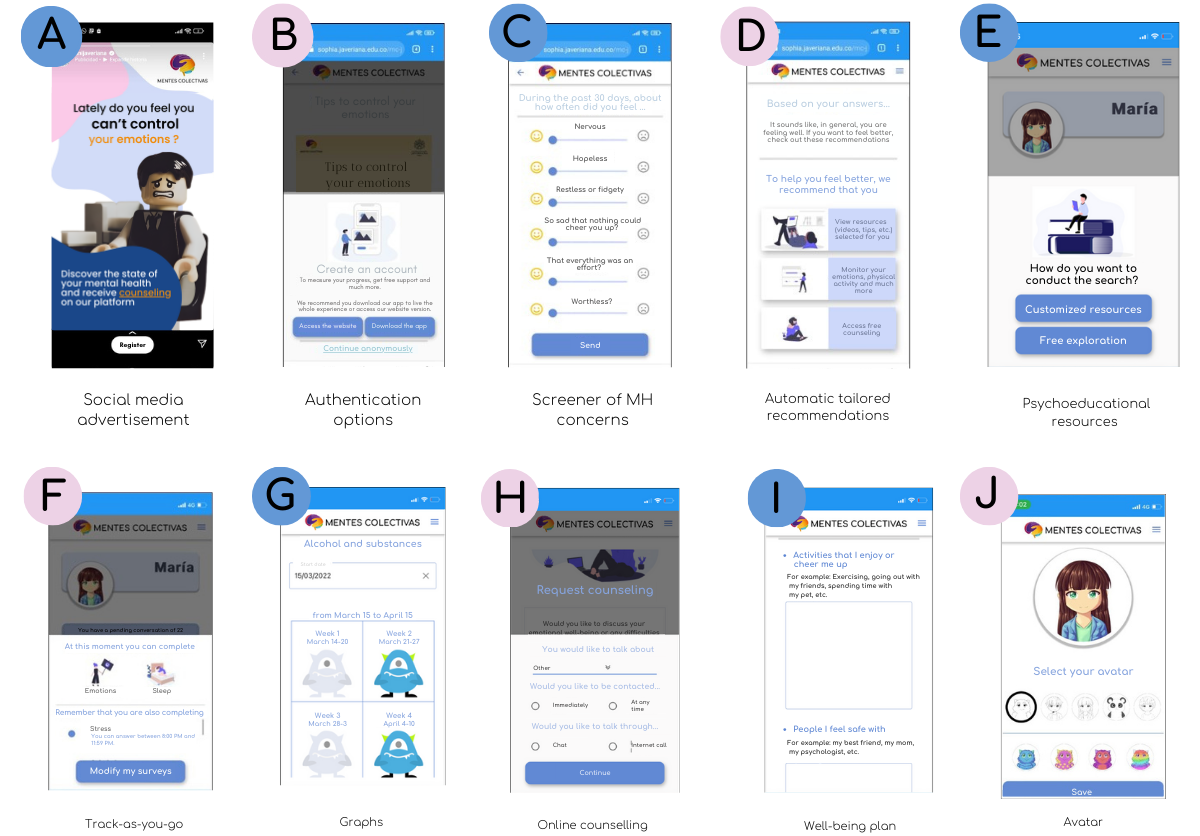
User journey of the platform. Platform screenshots were translated from Spanish to English for clarity. The app version of the platform is currently under development. MH: mental health.

Recognizing the importance of outreach, we developed a social media campaign featuring advertisements with cartoons to promote the platform and attract users (Figure 1). While awareness campaigns and influencer collaborations could further enhance visibility, they were not included at this stage. This initial approach allowed us to gauge user engagement and refine strategies for future promotional efforts.

##### From Well-Being Nudges to Well-Being Plan

To encourage young people to engage in MH-promoting activities, we aimed to incorporate well-being nudges throughout the platform. Some of these nudges were successfully integrated into the monitoring graphs, providing gentle prompts to encourage reflection and engagement. However, expanding nudges across the entire platform proved challenging.

As an alternative, we developed the well-being plan, a structured tool inspired by safety plans. It helps users identify uplifting activities, supportive people, and emotional warning signs. Users can personalize their plan with open-text responses and are encouraged to practice beneficial activities when facing challenges. The platform also provides examples of warning signs, such as weight loss or insomnia, ensuring accessibility at all times.

Given development constraints, we prioritized the Wellbeing Plan as the core feature for the MVP, with the potential for further integration of nudges and additional tools in future iterations.

##### Emergencies

To respond to this need, we designed the telecounseling feature to allow participants to speak with someone immediately from Monday to Friday, 7 AM to 7 PM. During off-hours, the button provided continuous access to emergency local services information (eg, gender-based violence and suicide-related Colombian helplines), ensuring support was always available. Given that the platform is web based, automatic dialing could not be integrated.

##### Deferred Functionalities

The remaining functionalities were not developed for this MVP due to resource constraints and implementation complexities. Community participation was deprioritized to ensure platform sustainability and focus on essential features such as virtual care, as moderation and content creation required dedicated budgets and personnel. Artificial intelligence functionalities were also not included due to technical challenges and safety concerns, as current large language models in Spanish required significant refinements to ensure reliability and user safety. Interoperability with local services was set aside due to the complexity of institutional agreements, data management, and privacy concerns. Finally, while research and public health surveillance were recognized as valuable, they fell outside the MVP’s scope, as they required advanced data management, administrative agreements, and rigorous validation for public health use. Future developments may reassess the feasibility of these features to enhance user experience and support services.

#### User Journey

The user journey (Figure 1) begins by attracting potential users through targeted advertisements on social media. When users click on an advertisement, they are redirected to the platform’s landing page, where they find general information about the platform, MH, and well-being. From there, they can choose to log in and create an account for full access or continue anonymously with limited functionality. If they opt to register, they are guided through a straightforward sign-up process where they provide informed consent, accept the platform’s terms and conditions, and acknowledge the habeas data regulations for data protection.

After completing registration, users take a screening test designed to assess their MH concerns. Once they finish the test, they immediately receive feedback on their results and are invited to explore targeted and specialized resources based on their responses. They can also start monitoring their MH through the track-as-you-go feature, which allows them to select areas of interest and continuously track them over time. On the basis of their tracking results, further specialized algorithms provide tailored recommendations and psychoeducational resources. Users can also request a web-based counseling session if needed.

Registered users can access additional features on their home page, including the SOS button, a customizable well-being plan, and avatar selection. As users continue engaging with the platform, they receive increasingly personalized information, whether by tracking additional areas or adjusting their preferences over time. This ensures that the platform evolves with their needs, offering continuous and adaptive support for their mental well-being.

#### Usability Results

A total of 36 individuals participated in usability sessions for the alpha prototype (n=16, 44%) and the beta prototype (n=20, 56%).

##### Task Completion Results

In alpha tests, the median task completion time was 43 (IQR 23.5-66.5) seconds, with a median score of 1.00 (IQR 1-1) on the Single Ease Question scale, showing minimal difficulty in performing tasks. The beta tests showed similar results, with a median completion time of 28 (IQR 16-48) seconds and a score of 1.00 (IQR 1-2; Table 2). The track-as-you-go surveys generally showed favorable results with short completion times, except for the physical activity test, which had the longest time and highest difficulty. Accessing resources, returning to profiles, and requesting telecounseling increased completion time without impacting the experience (Table 3).

The beta prototype had a System Usability Scale median score of 85.0 (IQR 80-92.5), indicating acceptable usability. Nearly all participants (18/20, 90%) found the monitoring surveys easy to use. Most (14/20, 70%) found the avatar functionality visually appealing, though some (7/20, 35%) suggested more customization options. The SOS feature was considered useful by all (14/20, 70%), with participants suggesting it should be made more visible.

Improvements between the alpha and beta builds mostly included corrections to the back end of the platform to solve bugs and reduce malfunctioning. Front-end improvements were limited to changes in the wording of instructions, feedback from test results, headings, or descriptions.

**Table 2:** Completion times and Single Ease Question (SEQ) scores for tasks conducted in alpha and beta user testing sessions. The number of participants who performed the task or responded to the SEQ is presented for each task, as time restrictions or platform malfunctioning impeded all participants from completing all activities^a^.

Task	Alpha test			Beta test	
	Completion time (s), median (IQR)	SEQ score, median (IQR)	Completion time (s), median (IQR)		SEQ score, median (IQR)
Creating an account	67.50 (47-89.75)^b^	1.00 (1-1)^b^	61.50 (53.25-91.75)^c^		1.00 (1-1)^c^
Filling the screener of concerns	43.00 (34.25-50.75)^b^	1.00 (2-1)^d^	34.00 (30.5-44.5)^c^		2.00 (3-1)^c^
Accessing MH^e^ resources	21.00 (15.25-70)^b^	1.00 (1-1.25)^f^	59.50 (35-83)^c^		1.00 (1-1)^c^
Going to my profile	5.00 (2.75-13)^f^	1.00 (1-1)^g^	6.00 (5-13)^h^		1.50 (1-3)^i^
Requesting a web-based counseling session	44.00 (39-72.5)^j^	1.00 (1-1.5)^j^	76.00 (64-101)^h^		1.00 (1-1)^i^

^a^Median values were used, considering the small samples, to provide a more accurate representation of central tendency.

^b^n=16.

^c^n=20.

^d^n=15.

^e^MH: mental health.

^f^n=14.

^g^n=11.

^h^n=19.

^i^n=18.

^j^n=13.

**Table 3:** Completion times and Single Ease Question (SEQ) scores for tasks conducted only in beta user testing sessions^a^.

Task		Completion time (s), median (IQR)	SEQ score, median (IQR)
Filling out the sociodemographic survey		11.00 (10-14.75)^b^	1.00 (1-1)^c^
**MH^d^ monitoring^e^**			
	Stress	23.00 (17–29)^c^	1.00 (1–1)^c^
	Loneliness	16.00 (13.75–24)^c^	1.00 (1.75–1)^c^
	Well-being	37.50 (29–49.75)^c^	1.00 (2–1)^f^
	Emotions	47.00 (32–63)^c^	1.00 (2–1)^c^
	Physical activity	51.50 (37.25–68)^c^	3.00 (1.25–5)^c^
	Sleep	18.50 (15–24.25)^c^	1.00 (2–1)^c^
	Subjective health status	16.00 (11.5–17.25)^g^	1.00 (1–1)^g^
	Self-esteem	26.50 (16.25–33.5)^c^	1.00 (3–1)^c^
	Relationships	30.00 (26.25–44.75)^c^	1.00 (2–1)^c^
	Eating	27.00 (20.25–34.75)^c^	1.00 (1–1)^c^
	Alcohol and substance use	35.00 (29.25–41.5)^c^	1.00 (1–1)^c^
	Bullying	10.50 (9–15)^c^	1.00 (1–1)^c^
Filling the well-being plan		102.00 (149-86.5)^h^	2.00 (1-2)^h^
Using the SOS button		15.50 (26.5-10)^i^	1.00 (1-1)^i^

^a^Median values were used, considering the small samples, to provide a more accurate representation of central tendency.

^b^n=20.

^c^n=20.

^d^MH: mental health.

^e^During beta sessions, all MH monitoring surveys that the platform offered were tested. An additional test (sexual and reproductive health) was developed after beta sessions had concluded and thus is not included in this table.

^f^n=19.

^g^n=18.

^h^n=13.

^i^n=14.

##### Reliable Information

Participants (13/16, 81%) from alpha user testing sessions found the topics of the tips useful and relevant. Furthermore, they (12/16, 75%) liked the images and tips design and described them as striking and pleasant. In total, 12% (2/16) of the participants believed the tips were generic or random, and 6% (1/16) of them found the design boring. In the beta sessions, participants (16/20, 80%) expressed their satisfaction with the tips and their content. However, most of them (16/20, 80%) suggested design changes, including color and image updates, and adding more information to the tips by providing links to additional resources. Tips were not accepted by 15% (3/20) of the participants who did not find them useful and suggested changes to the text and more detailed explanations.

##### Registration

Most (14/16, 88%) of the participants from the alpha testing sessions found the account creation process easy. Only 1 participant was confused by the registration process. In terms of registration options, 31% (5/16) of the participants favored the web version, 31% (5/16) preferred the app version, 12% (2/16) opted for the anonymous route, while the rest (4/16, 25%) did not express any preferences. Most participants (14/16, 88%) understood the sociodemographic questions, and more than half (11/16, 69%) found the images and questions design aesthetically pleasing. The others suggested making changes, especially regarding the gender question and the text description of the platform. Moreover, 19% (3/16) of the participants thought that the survey should collect information about their motive for consultation. The platform’s description was changed based on feedback; however, the motive for consultation was not included due to the platform’s focus on promotion and prevention, and no changes were made to the gender questions due to limitations in illustration availability.

In the beta sessions, 9 (45%) of the 20 participants preferred logging in to the platform through the web, and 6 (30%) of the 20 participants chose to access the platform anonymously. All participants (20/20, 100%) found the survey easy and clear and considered the gender question to be acceptable, inclusive, and noninvasive. They made some recommendations related to including more questions or additional answer options.

##### Virtual Care

In alpha user testing sessions, all participants (13/13, 100%) were able to easily access the feature. They valued its shortness and simplicity but suggested adding more MH topics in the request form. A participant stated that requesting a session involved too many steps. Due to parameterization issues, no topics were added to the request form. In beta user testing sessions, almost all interviewees (18/19, 95%) liked the web-based counseling functionality for its usefulness and flexibility.

##### Screening Tests and Continuous Monitoring of MH Areas

In alpha user testing, 62% (10/16) of the participants found the screener test easy but suggested adding instructions and more intermediate options. Most (11/16, 69%) participants understood the recommended functionalities, though 25% (4/16) of the participants had trouble with the monitoring feature. Most (12/16, 75%) participants had no issues completing surveys or sharing information but suggested improving the design, adding options to the emotions survey, and making the survey list more prominent. For the beta build, instructions were revised, but intermediate options were excluded to preserve consistency with the original scales. The appearance of the feature on the main profile was also revised, changing the wording of the title to increase clarity.

In beta sessions, nearly all participants (18/20, 90%) liked the screener of concerns, finding it easy, useful, and interactive. They suggested changes to the visibility of the scale, such as making the slider larger, making the answer options visible, or adding instructions (13/20, 65%). More than half of the interviewees (12/20, 60%) understood the 3 recommendations given after the test, with the monitoring feature recommendation being the least understood (7/20, 35%). Nearly all of the participants (18/20, 90%) found most surveys easy to use, and the feedback was positive overall. The most difficult survey was physical activity (6/20, 30%), where participants had trouble making estimates in minutes. They suggested changing the survey to ask about hours; however, due to parameterization restrictions, it was not possible to accommodate this request. Participants also recommended including more targeted resources and additional answer options for the emotions, bullying, and self-esteem surveys, among others. To comply with these recommendations, changes would have to be made to algorithms, which require investments in time and resources. Thus, this recommendation will be considered for further developments. Graphs were also tested in beta sessions, where the calendar type with icons was the most accepted. Participants suggested changes to the icons for most of the graphs, especially the well-being graph (12/20, 60%), followed by the loneliness graph (10/20, 50%).

##### Gamification

To prioritize other functionalities, the avatar selection feature was only tested in beta sessions. Most of the participants (14/18, 78%) found it visually appealing. Some of them (7/18, 39%) suggested more options, such as color customization and adding more avatar types. In total, 1 participant did not like the anime style.

##### Integration With Social Media and Advertising

Alpha user testing participants (9/16, 56%) suggested changes in the image and colors to make the advertising more striking and more related to the MH topic. Among the options, the most liked one used cartoons (12/20, 60%) instead of pictures. This advertisement was used in beta sessions where slightly more than half of the participants (13/20, 65%) found it appealing.

##### Well-Being Nudges

The well-being plan was tested in beta sessions. Participants liked the tool and found it useful and easy to manage. They suggested adding examples of signs of distress or default options to choose from.

##### Emergencies

The SOS feature was only tested in beta sessions, where all participants (14/14, 100%) found it useful. They valued the variety of helplines and contact options and suggested making it more visible in the main profile.

### Testing

Although the platform was designed for young people, feedback from CDWs indicated interest from support individuals, such as parents, caregivers, or health professionals, who expressed a desire to explore the platform before their children or patients used it. In response, we decided to collect demographic information from all users, specifying their role or profile. During the naturalistic trial, no age restrictions were imposed, allowing us to analyze data from all users, including adults, as part of the platform evaluation.

A total of 452 individuals entered the platform. Due to technical difficulties (and hence incomplete information), 17 (3.8%) records were excluded from the analysis. There were 3 registration peaks: the first in November 2022, the second in March 2023, and the third in June 2023. The platform reached 26 of the country’s 32 departments; most of the users were from Bogotá (139/314, 44.3%), Cundinamarca (25/314, 8%), Boyacá (23/314, 7.3%), and Antioquia (15/314, 4.8%). During the observed period, 435 individuals used the platform, with 314 (72.2%) users registering and 121 (27.8%) using it anonymously. Most anonymous users were female (89/121, 73.6%) as well as identified to be a young person (31/121, 25.6%). Importantly, more than half (74/121, 61.2%) of the users presented some degree of emotional distress according to the 6-item Kessler Psychological Distress scale (mild distress: 29/121, 24%; moderate distress: 33/121, 27.3%; and severe distress: 12/121, 9.9%) [32].

In relation to those who registered, the median age was 34 (IQR 23-46) years, and most of them (232/314, 73.9%) were female. About the role in the platform, 21% (66/314) of those who registered were young individuals, 26.8% (84/314) were carers, 22% (69/314) were educators, and 20.7% (65/314) were health professionals. Most users (200/314, 63.7%) presented some degree of emotional distress (mild distress: 68/314, 21.7%; moderate distress: 70/314, 22.3%; and severe distress: 62/314, 19.7%).

To evaluate the relationship between the level of distress and the selected role, a chi-square test of independence was executed. In the registered group, the relationship was statistically significant (*χ*²_9_=37.9; *P*<.001), and observed frequencies revealed that young individuals had more severe, moderate, and mild levels of distress, while health professionals appeared more frequently with low levels of distress. In addition, an effect size was calculated using Cramér V, which was found to be 0.20. While this effect size is statistically significant, it is small in magnitude. In the group of anonymous users, the relationship was statistically significant (*χ*²_9_=17.0; *P*=.048), and the Cramér V test showed a weak effect (0.22). The observed frequencies showed that young people had more severe levels of distress, carers and young people had more moderate levels of distress, carers and educators had more mild levels of distress, and health professionals had lower levels of distress more frequently.

Regarding user engagement, 81.2% (255/314) of the individuals used the platform for just 1 day, 8.9% (28/314) used it for 15 days, 2.5% (8/314) used it for 1 month, and 7.3% (23/314) used it for over a month up to 1 year. Among the functionalities, the screening test (314/314, 100%) was the most used as it was mandatory, followed by track-as-you-go (118/314, 37.6%), telecounseling request (102/314, 32.5%), well-being plan (32/314, 10.2%), SOS button (15/314, 4.8%), and finally avatar (14/314, 4.5%). A chi-square test of independence was executed between the level of distress and the platform’s use. The relationship was statistically significant (*χ*²_12_=24.5; *P*=.02), and the effect size was calculated using Cramér V, which was found to be 0.16, which meant a weak effect size. The observed frequencies indicated that people who only used the platform on the day of registration were more likely to report no discomfort in the emotional distress screening. In addition, severe discomfort was the most common outcome for people who used the platform during their first month of registration. People who used the platform for 2 months or between 3 and 6 months most often experienced moderate discomfort. By contrast, those who used the platform for >6 months had higher scores in severe and mild discomfort.

Among the individuals who used the tracking component, the most frequently used survey was the emotion tracker (32/118, 27.1%), followed by sleep (25/118, 21.2%), stress (21/118, 17.8%), and physical activity (10/118, 8.5%). The rest of the surveys (loneliness feeling: 6/118, 5.1%; well-being levels: 6/118, 5.1%; self-esteem evaluation: 5/118, 4.2%; risk of eating disorders: 4/118, 3.4%; health perception: 3/118, 2.5%; sexual health: 3/118, 2.5%; relationships: 2/118, 1.7%; and bullying: 1/118, 0.8%) represented less than 5% use. In addition, only a small number of participants opted to continuously monitor any of these areas, with 47% (15/32) tracking their emotions, 44% (11/25) tracking sleep, 24% (5/21) tracking stress, and 20% (2/10) tracking physical activity beyond the initial use.

Concerning the baseline (first time) responses, neutral emotion was the most reported with 41% (13/32), followed by sadness 22% (7/32), fear 19% (6/32), anger 12% (4/32), and joy 6% (2/32). In terms of sleep quality, 96% (24/25) of the users who answered the sleep survey reported sleep problems. With respect to the perceived stress, 71% (15/21) of the participants experienced high levels of stress, 24% (5/21) had some degree of stress, and 5% (1/21) had low stress. In relation to physical activity, participants reported spending a median of 10 (IQR 0-37.5) minutes doing moderate activity per day, with just 10% (2/10) of the users doing it for >60 minutes daily. Median sitting time was 210 (IQR 47.5-345) minutes.

Of the 102 participants who requested telecounseling, only 27 (26.5%) had a complete counseling session. Regarding the reasons for consulting, the most frequent problem was anxiety, reported by 67% (18/27) of the participants, followed by sadness at 11% (3/27), and everyday problems at 11% (3/27). In terms of the form of communication, the platform offered 5 types: immediate chat, chat later, immediate internet call, internet call later, and video call and traditional phone call. Chat later and immediate call options were the main forms chosen by users, with 48% (13/27) and 33% (9/27), respectively. Moreover, the immediate chat option and internet call me later option were less popular among participants, with only 19% (5/27) and 15% (4/27). Finally, video call (1/27, 4%) was the least used option. During the counseling session, only 4% (1/27) of the individuals reported self-harm and suicide plans, which required referral to emergency services.

## Discussion

### Principal Findings

This study aimed to co-design, develop, and test an MH platform tailored for youth in Bogotá, Colombia. Using a rigorous R&D cycle, we actively engaged a diverse sample, ensuring representation across different ages, socioeconomic backgrounds, and marginalized or hard-to-reach populations. Findings revealed a strong interest in a youth-focused MH platform that prioritized a visually appealing and user-friendly interface, clear privacy protections, and a range of essential features. These included registration, access to reliable information, virtual care options, MH screening and continuous monitoring, gamification, social media integration, personalized well-being plans, emergency support, connections to local services, chatbot interaction, and support for research and public health surveillance. Through knowledge translation sessions, we refined these insights into a platform with 11 key features, including social media integration, registration, MH screening tools, psychoeducational resources, automated recommendations, real-time MH tracking across 13 areas, follow-up graphs, telecounseling integration, customizable well-being nudges, an emergency button, and gamification components. Usability testing confirmed high functionality and acceptance, and a naturalistic trial further evaluated real-world engagement and use patterns. This extensive participatory approach not only ensured that the platform was contextually relevant and user driven but also demonstrated its acceptability, usability, and potential impact in supporting youth MH.

A notable aspect of our research is the extensive engagement of end users throughout the co-design process, with a sample size of 146 participants. Existing literature indicates that most studies using participatory methodologies in the development of MH DHTs are conducted in high-income countries and often involve relatively small sample sizes (<50 participants). In Latin America, a unique instance of co-design was conducted in Colombia, featuring culturally adapted approaches [24]. Despite the inclusive methodology, men and other genders represented <30% of the sample. This level of participation aligns with research showing that men tend to seek less MH support [33] and engage less in research [34]. This disparity highlights a research gap in understanding the engagement of men and gender-diverse individuals in MH initiatives, which may also impact their adoption and use of DHTs [16]. Future work is needed to address this gap and ensure these technologies are truly inclusive and accessible to all [16,35].

Moreover, the ample sample size of this study allowed for the inclusion of individuals who may act as gatekeepers in young people’s MH help-seeking journeys, offering valuable insights into how their involvement can support youth. In addition, the study captured a broad spectrum of perspectives, including diverse and sometimes conflicting opinions, not only regarding design preferences but also in the roles and expectations of caregivers versus young people. These conflicting perspectives highlight a fundamental tension in designing DHTs, that is, balancing the autonomy and preferences of young users with the concerns and involvement of caregivers. However, this diversity of opinions enriches the research process by prompting critical discussions and leading to more adaptable, inclusive, and responsive solutions. Future work should further explore these tensions and develop strategies to ensure that DHTs effectively support both young users and their caregivers while respecting youth autonomy and privacy [36].

This paper provides a comprehensive description of the participatory processes, thereby enriching the literature, as many studies do not provide comprehensive details, thus limiting the reader’s understanding of the methodologies used and the extent of user involvement [37]. Our study sought to provide detailed information on the participation of end users at every stage of the development process, including design, feedback provision, knowledge translation, decision-making, and testing. These results highlight the need for end users to be at the center of the design and implementation process rather than just being mere consultants [22,23]. In the same line, the process involved young people and people with lived experience in a meaningful way, avoiding tokenism and promoting genuine participation [38].

In terms of use, engagement is a significant challenge in the eHealth field, with research consistently showing high attrition and low engagement rates [11]. For example, studies indicate that retention rates for mental well-being apps are as low as 3.9% after 15 days and 3.3% after 30 days [39]. In our study, we observed a particularly striking trend on the first day of use, with only 18.8% (59/314) of the users continuing to engage with the platform after the first day. Understanding and improving the first-day experience is crucial for enhancing overall retention and ensuring users continue to benefit from the platform. In our study, participants with higher levels of distress demonstrated a tendency to use the platform for extended periods, aligning with existing evidence [40]. This suggests that the platform may be particularly well-suited for individuals already engaged in the MH care process. Engagement remains a key area for future R&D to address the substantial drop-off after initial use. While our user engagement rates were better (longer periods of engagement) than those reported in previous research, possibly due to our thorough co-design process tailored to users’ needs and preferences, achieving long-term engagement requires sustained efforts and innovative strategies to keep users motivated and invested over time. Some alternatives were highlighted by our participants, which referred to integration with local services (in person or virtual), which are already available in Colombia and are known to communities. This synergistic approach could provide more customized follow-ups for service users while expanding the platform’s reach to communities outside of social media, creating a blended approach to engagement. However, integration represents a challenge as it requires coordinated responses as well as administrative and data protection agreements.

Our study aimed to contribute to reducing the significant delay in seeking help for MH problems, which averages >10 years [6] from the first symptom to professional contact. By implementing our platform, we provide a potential tool to facilitate earlier access to support, addressing the urgent need for more accessible and timely MH interventions. Internet-based interventions have shown promise in this regard, offering a cost-effective alternative to traditional care [16]. A meta-analysis indicated that these interventions are marginally more effective than usual care in terms of quality-adjusted life years gained, have similar prices, and have a net benefit of US $255 (95% CI US $91-US $419; *P*=.002) [41]. Furthermore, research has shown that targeted prevention efforts are generally more cost-effective than universal approaches in the youth population [42]. Specifically, interventions that are deployed in community settings (eg, schools or workplaces) and coupled with specific interventions (eg, psychotherapy) have proven highly cost-effective for preventing MH disorders in children and adolescents [42].

Our findings support the need for rethinking services and care pathways, as two-thirds of the users (both anonymous and registered) showed some level of emotional distress. Young users exhibited higher distress levels, with a notable relationship between distress levels and user roles (*χ*²_9_=37.9; *P*<.001) as demonstrated in the Results section. These insights emphasize the potential of our platform to identify individuals in need of immediate support. By integrating such technologies with traditional MH services, we can enhance early detection and intervention, ultimately improving MH outcomes. Our findings advocate for the continued innovation, development, and implementation of DHT to detect early signs of distress and integrate these tools with conventional care systems to better support young people and those at risk early in the course of illness.

### Limitations

In academia, it is difficult to compete with commercial DHTs that offer highly intuitive and engaging user experiences, even though their content often lacks solid evidence-based support [43,44]. A significant limitation of our study is the absence of collaboration with industry partners, which could provide valuable resources and expertise in user-centered design and technology development at commercial standards. Partnerships that leverage industry expertise could result in more usable and high-fidelity technologies [44] while also facilitating improved data collection methods, such as the use of phone sensors or wearable devices, to enhance data accuracy and enable more timely notifications, advancing to precision medicine [45]. Furthermore, partnerships with industry could help address the critical challenges of innovation, sustainability, and scalability. Moving forward, it is essential to combine rigorous evidence-based methods with the engaging design features typical of commercial products, along with the advantages provided by industry collaboration, to develop effective, sustainable, and scalable digital health interventions.

### Implementation in the Colombian Context

Although participants reported good acceptability of the platform, there are several barriers to its implementation in the Colombian context. A key issue is the discrepancy between young people’s needs and legal requirements. Colombian regulations mandate parental consent for access to most health services [46]. While some young people were willing to involve their guardians, most preferred using the platform independently. Parents, while desiring access to their children’s information, recognized this might deter openness. To address this, an anonymous browsing option was created, requiring registration only for specialized functions, such as receiving targeted information or telecounseling.

The co-design process was conducted in Bogotá, which, though diverse, may not represent all populations. Further adaptations are needed to tailor the platform to the MH needs of different Colombian communities, including minority groups.

### Conclusions

In conclusion, this study successfully co-designed and tested an MH platform tailored for youth in Bogotá, Colombia, to promote help-seeking behaviors for MH issues. Engaging a diverse sample of 146 participants from various backgrounds, the platform addressed the preferences of users across different ages and socioeconomic statuses, demonstrating high acceptability and usability. Unlike many studies with smaller sample sizes, our inclusive methodology underscored the importance of involving end users throughout the development process. However, engagement remains a challenge, with only 18.8% (59/314) of the users continuing to engage with the platform after the first day. Future work must enhance the initial user experience and consider differences in age, sex, region, and socioeconomic background to improve long-term engagement.

Despite the promising results, a significant limitation was the lack of collaboration with industry partners, which could provide valuable resources and expertise in user-centered design and technology development. Partnerships could improve data collection methods, address challenges of sustainability and scalability, and integrate rigorous evidence-based methods with engaging design features. While the platform was well received, implementing it in the Colombian context presents challenges, such as legal requirements for parental consent, which can deter young people from seeking help. Further research and adaptations are needed to tailor the platform to the unique characteristics and MH needs of diverse Colombian communities, ensuring it effectively supports early detection and intervention.

## Abbreviations

CDW: co-design workshop
CF: coding frequency
DHT: digital health tool
MH: mental health
MVP: minimum viable product
R&D: research and development
REDCap: Research Electronic Data Capture

## Data Availability

The datasets generated or analyzed during this study are available from the corresponding author on reasonable request.
